# Drugs that simultaneously down-regulate IL-1β and TNF secretion significantly reduce inflammation in a rat model of antigen-induced arthritis

**DOI:** 10.1186/1479-5876-9-S2-P21

**Published:** 2011-11-23

**Authors:** Rita Cascão, Bruno Vidal, Helena Raquel, Vineet Gupta, João E Fonseca, Luis F Moita

**Affiliations:** 1 Instituto de Medicina Molecular, Faculdade de Medicina da Universidade de Lisboa, Lisbon, Portugal; 2 Division of Nephrology and Hypertension, Dept. of Medicine, University of Miami, Miami, Florida, USA; 3 Rheumatology Dept., Centro Hospitalar de Lisboa Norte, EPE, Hospital de Santa Maria, Lisbon, Portugal

## Introduction

We have previously reported an increase in interleukin (IL)-1β levels and a continuous activation of caspase-1 in rheumatoid arthritis (RA) patients in the early phase of the disease. This result suggests that drugs targeting IL-1β regulatory pathways, in addition to tumor necrosis factor (TNF), may constitute promising therapeutic agents in early RA. We have recently used a THP-1 macrophage-like cell line to screen 2320 compounds for those which down-regulate IL-1β and TNF secretion. Gambogic acid, celastrol and pristimerin were three of the most promising therapeutic candidates identified in that study.

## Aim

Our main goal here is to investigate whether administration of gambogic acid, celastrol or pristimerin is able to attenuate inflammation in a rat model of antigen-induced arthritis (AIA).

## Methods

Drugs were administered to AIA rats (N=5-10 per group) in the early phase of arthritis, after 4 days of disease induction and for a period of 15 days. The inflammatory score, paw perimeter and body weight were evaluated during the period of treatment. Rats were sacrificed after 19 days of disease evolution and paw samples were collected for histological and immunohistochemical evaluation. Moreover, in vitro studies were performed with THP-1 macrophage-like cell line cultured with each drug, and the levels of caspase-1 and NF-kB activation were measured.

## Results

We found that both gambogic acid and celastrol significantly suppressed inflammation in joints. The histological and immunohistochemical evaluation revealed that treated rats had a normal joint structure with complete abrogation of the inflammatory infiltrate and prevention of synovial cells proliferation. Furthermore, we observed that gambogic acid and celastrol inhibit caspase-1 and NF-kB activation.

## Conclusions

Our results showed that treatment with gambogic acid and celastrol protected AIA rats from arthritis development with a complete abrogation of joint immune cellular infiltration, proliferation and prevention of cartilage and bone damage. In conclusion, gambogic acid and celastrol can putatively constitute anti-inflammatory drugs with therapeutic efficacy in the treatment of inflammatory diseases such as RA, possibly through the down-regulation of caspase-1 and NF-kB activation.

